# Dichloridobis(3,4,5-trimethyl-1*H*-pyrazole-κ*N* ^2^)cobalt(II)

**DOI:** 10.1107/S160053681003254X

**Published:** 2010-08-21

**Authors:** Ganna Lyubartseva, Sean Parkin

**Affiliations:** a Department of Chemistry and Physics, College of Science and Technology, Southern Arkansas University, Magnolia, AR 71753, USA; b Department of Chemistry, University of Kentucky, Lexington, KY 40506, USA

## Abstract

In the title compound, [Co^II^Cl_2_(C_6_H_10_N_2_)_2_], a pair of 3,4,5-trimethyl­pyrazoles act as monodentate ligands. Two Cl^−^ anions are also bonded directly to the Co^II^ atom, which has a CoN_2_Cl_2_ chromophore in a slightly distorted tetra­hedral geometry. The two mol­ecules in the asymmetric unit are related by an approximate twofold rotation roughly parallel to the *a* axis. The amino H atom in the pyrazole ring participates in weak N—H⋯Cl hydrogen bonds to form chains that propagate roughly parallel to the *c* axis.

## Related literature

For a similar tetra­hedral complex with pyrazole, see: Zyryanova *et al.* (2005 ▶). For thermal decomposition studies, see: Petrovic *et al.* (1993 ▶). For a similar tetra­hedral complex with 3,5-dimethyl­pyrazole, see: Leovac *et al.* (2007 ▶). For potential catalytic applications, see: Li *et al.* (2009 ▶); Oki *et al.* (1995 ▶). For additional related complexes, see: Sheu *et al.* (1996 ▶); Lyubartseva & Parkin (2010 ▶).
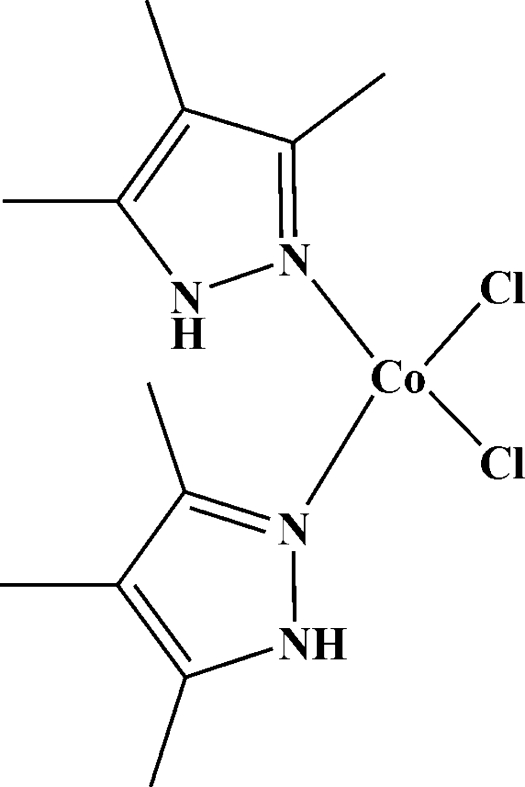


## Experimental

### 

#### Crystal data

[CoCl_2_(C_6_H_10_N_2_)_2_]
*M* *_r_* = 350.15
Orthorhombic, 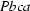

*a* = 14.8880 (1) Å
*b* = 17.3980 (1) Å
*c* = 24.9220 (2) Å
*V* = 6455.33 (8) Å^3^
*Z* = 16
Mo *K*α radiation
μ = 1.39 mm^−1^
*T* = 90 K
0.30 × 0.28 × 0.19 mm

#### Data collection

Nonius KappaCCD diffractometer
Absorption correction: multi-scan (*SCALEPACK*; Otwinowski & Minor, 1997 ▶) *T*_min_ = 0.590, *T*_max_ = 0.746
111609 measured reflections
7404 independent reflections
5822 reflections with *I* > 2σ(*I*)
*R*_int_ = 0.064

#### Refinement

*R*[*F* ^2^ > 2σ(*F* ^2^)] = 0.043
*wR*(*F* ^2^) = 0.111
*S* = 1.15
7404 reflections
355 parameters
H-atom parameters constrained
Δρ_max_ = 0.80 e Å^−3^
Δρ_min_ = −0.60 e Å^−3^

### 

Data collection: *COLLECT* (Nonius, 1998 ▶); cell refinement: *SCALEPACK* (Otwinowski & Minor, 1997 ▶); data reduction: *DENZO-SMN* (Otwinowski & Minor, 1997 ▶); program(s) used to solve structure: *SHELXS97* (Sheldrick, 2008 ▶); program(s) used to refine structure: *SHELXL97* (Sheldrick, 2008 ▶); molecular graphics: *XP* in *SHELXTL* (Sheldrick, 2008 ▶); software used to prepare material for publication: *SHELXL97* and local procedures.

## Supplementary Material

Crystal structure: contains datablocks global, I. DOI: 10.1107/S160053681003254X/ng5017sup1.cif

Structure factors: contains datablocks I. DOI: 10.1107/S160053681003254X/ng5017Isup2.hkl

Additional supplementary materials:  crystallographic information; 3D view; checkCIF report

## Figures and Tables

**Table 1:** Hydrogen-bond geometry (Å, °)

*D*—H⋯*A*	*D*—H	H⋯*A*	*D*⋯*A*	*D*—H⋯*A*
N2*A*—H2*A*⋯Cl2*B*^i^	0.88	2.55	3.290 (2)	142
N2*A*—H2*A*⋯Cl2*A*	0.88	2.86	3.373 (2)	118
N4*A*—H4*A*⋯Cl1*B*^ii^	0.88	2.61	3.416 (2)	152
N4*A*—H4*A*⋯Cl1*A*	0.88	2.79	3.309 (2)	119
N2*B*—H2*B*⋯Cl2*A*^iii^	0.88	2.70	3.456 (2)	145
N2*B*—H2*B*⋯Cl2*B*	0.88	2.76	3.286 (2)	119
N4*B*—H4*B*⋯Cl1*A*^iv^	0.88	2.57	3.363 (2)	150
N4*B*—H4*B*⋯Cl1*B*	0.88	2.86	3.365 (2)	118

Symmetry codes: (i) 
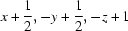
; (ii) 
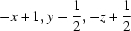
; (iii) 
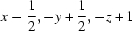
; (iv) 
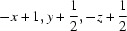
.

**Fig. 1.**
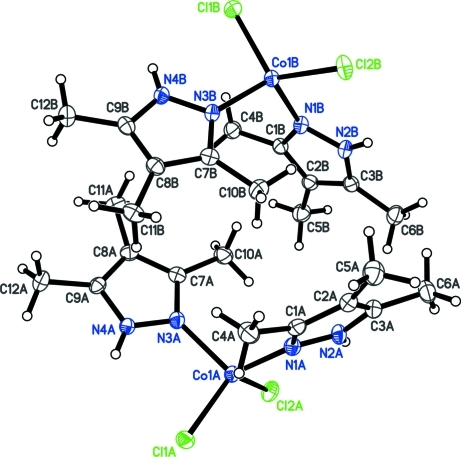
View of the title compound with the atom numbering scheme. Displacement ellipsoids are drawn at the 50% probability level.

## supplementary crystallographic information

### Comment

Pyrazole complexes with transition metals are very important because of their similarities to regular constituents of proteins. They can also be potentially useful for catalysis (Li *et al.* 2009) and magnetism studies (Oki *et al.* 1995). To obtain a tetrahedral complex geometry it is often necessary to engineer the pyrazole ligand with different connectivity (Sheu *et al.* 1996). Here we report that the introduction of one more methyl group in the pyrazole ring at 4 position, in addition to 3 and 5 position, results in the efficient formation of a tetrahedral geometry.

The synthesis and characterization of the cobalt co-ordination compound containing the 3,4,5-trimethylpyrazole ligand is described. The compound has the formula Co^II^(C_6_H_10_N_2_)_2_Cl_2_ and been characterized by several spectroscopic methods and analytical techniques. 3,4,5-Trimethylpyrazole acts as a monodentate ligand and the anions are also bonded directly with the metal center. The cobalt atom has a CoN_2_Cl_2_ chromophore in a distorted tetrahedral geometry. Co–N distances are 2.004 (2) and 2.012 (2)Å for the pyrazole N atoms. Co–Cl distances are 2.2536 (7) and 2.2617 (7) Å. The amine hydrogen in the pyrazole ring participates in weak N—H—Cl hydrogen bonds to form chains that propagate roughly parallel to the *c* axis direction.

### Experimental

CoCl_2_.6H_2_O (0.714 g, 3 mmol) was added to 3,4,5-trimethylpyrazole (0.668 g, 6 mmol) in a 50 ml round bottom flask and 20 ml tetrahydrofuran added to the reaction mixture with stirring. After 5 minutes stirring, the blue color reaction mixture was filtered and the filtrate was added drop wise to 500 ml of hexane with vigorous stirring. The resulting powder was filtered and found to be [bis(3,4,5-trimethylpyrazole)]-dichlorocobalt(II) (0.698 g, Yield=66.4%). For structure determination, this powder was dissolved in methylene chloride and layerd with hexane. Blue colored, analytically pure orthorhombic crystals were obtained after 3 days. Elemental analysis calculated for CoC_12_H_20_N_4_Cl_2_: C 41.16, H 5.76, N 16.00; found C 41.11, H 5.69, N 16.08. IR(cm^-1^) 3310, 2925, 1581, 1530, 1446, 1404, 1377, 1266, 1199, 1159, 1125, 1018, 950, 765, 697, 683, 598, 566, 490.

### Refinement

H atoms were found in difference Fourier maps and subsequently placed in idealized positions with constrained distances of 0.98 Å (RCH_3_), 0.88 Å (N—H), and with *U*_iso_(H) values set to either 1.2*U*_eq_ or 1.5*U*_eq_ (RCH_3_) of the attached atom.

### Crystal data

**Table:**

[CoCl_2_(C_6_H_10_N_2_)_2_]	*F*(000) = 2896
*M**_r_* = 350.15	*D*_x_ = 1.441 Mg m^−^^3^
Orthorhombic, *P**b**c**a*	Mo *K*α radiation, λ = 0.71073 Å
Hall symbol: -P 2ac 2ab	Cell parameters from 15242 reflections
*a* = 14.8880 (1) Å	θ = 1.0–27.5°
*b* = 17.3980 (1) Å	µ = 1.39 mm^−^^1^
*c* = 24.9220 (2) Å	*T* = 90 K
*V* = 6455.33 (8) Å^3^	Block, blue
*Z* = 16	0.30 × 0.28 × 0.19 mm

### Data collection

**Table:**

Nonius KappaCCD diffractometer	7404 independent reflections
Radiation source: fine-focus sealed tube	5822 reflections with *I* > 2σ(*I*)
graphite	*R*_int_ = 0.064
Detector resolution: 9.1 pixels mm^-1^	θ_max_ = 27.5°, θ_min_ = 1.6°
ω scans at fixed χ = 55°	*h* = −19→19
Absorption correction: multi-scan (*SCALEPACK*; Otwinowski & Minor, 1997)	*k* = −22→22
*T*_min_ = 0.590, *T*_max_ = 0.746	*l* = −32→32
111609 measured reflections	

### Refinement

**Table:**

Refinement on *F*^2^	Primary atom site location: structure-invariant direct methods
Least-squares matrix: full	Secondary atom site location: difference Fourier map
*R*[*F*^2^ > 2σ(*F*^2^)] = 0.043	Hydrogen site location: inferred from neighbouring sites
*wR*(*F*^2^) = 0.111	H-atom parameters constrained
*S* = 1.15	*w* = 1/[σ^2^(*F*_o_^2^) + (0.0507*P*)^2^ + 7.0674*P*] where *P* = (*F*_o_^2^ + 2*F*_c_^2^)/3
7404 reflections	(Δ/σ)_max_ = 0.001
355 parameters	Δρ_max_ = 0.80 e Å^−^^3^
0 restraints	Δρ_min_ = −0.60 e Å^−^^3^

### Special details

**Table:**

Geometry. All e.s.d.'s (except the e.s.d. in the dihedral angle between two l.s. planes) are estimated using the full covariance matrix. The cell e.s.d.'s are taken into account individually in the estimation of e.s.d.'s in distances, angles and torsion angles; correlations between e.s.d.'s in cell parameters are only used when they are defined by crystal symmetry. An approximate (isotropic) treatment of cell e.s.d.'s is used for estimating e.s.d.'s involving l.s. planes.
Refinement. Refinement of *F*^2^ against ALL reflections. The weighted *R*-factor *wR* and goodness of fit *S* are based on *F*^2^, conventional *R*-factors *R* are based on *F*, with *F* set to zero for negative *F*^2^. The threshold expression of *F*^2^ > 2σ(*F*^2^) is used only for calculating *R*-factors(gt) *etc*. and is not relevant to the choice of reflections for refinement. *R*-factors based on *F*^2^ are statistically about twice as large as those based on *F*, and *R*- factors based on ALL data will be even larger.

### Fractional atomic coordinates and isotropic or equivalent isotropic displacement parameters (Å^2^)

**Table:**

	*x*	*y*	*z*	*U*_iso_*/*U*_eq_	
Co1A	0.70074 (2)	0.11088 (2)	0.365286 (14)	0.01780 (10)	
Cl1A	0.73843 (5)	0.00580 (4)	0.31744 (3)	0.02255 (15)	
Cl2A	0.80296 (5)	0.16183 (4)	0.42223 (3)	0.02503 (15)	
N1A	0.59573 (15)	0.09270 (13)	0.41394 (9)	0.0205 (5)	
N2A	0.59238 (15)	0.12310 (13)	0.46421 (8)	0.0211 (5)	
H2A	0.6375	0.1468	0.4799	0.025*	
N3A	0.66803 (15)	0.18364 (13)	0.30568 (8)	0.0196 (5)	
N4A	0.66770 (14)	0.16085 (12)	0.25323 (8)	0.0179 (4)	
H4A	0.6812	0.1142	0.2423	0.021*	
C1A	0.51360 (18)	0.06308 (14)	0.40500 (10)	0.0192 (5)	
C2A	0.45852 (19)	0.07446 (15)	0.44974 (10)	0.0218 (6)	
C3A	0.51108 (19)	0.11215 (15)	0.48690 (11)	0.0223 (6)	
C4A	0.49183 (19)	0.02581 (17)	0.35250 (11)	0.0268 (6)	
H4A1	0.4978	0.0636	0.3236	0.040*	
H4A2	0.4301	0.0063	0.3534	0.040*	
H4A3	0.5334	−0.0169	0.3462	0.040*	
C5A	0.36239 (19)	0.05170 (19)	0.45728 (12)	0.0306 (7)	
H5A1	0.3534	0.0335	0.4941	0.046*	
H5A2	0.3472	0.0105	0.4320	0.046*	
H5A3	0.3236	0.0962	0.4506	0.046*	
C6A	0.4881 (2)	0.13878 (18)	0.54196 (11)	0.0290 (6)	
H6A1	0.5432	0.1525	0.5612	0.044*	
H6A2	0.4570	0.0975	0.5613	0.044*	
H6A3	0.4488	0.1839	0.5397	0.044*	
C7A	0.64302 (17)	0.25790 (15)	0.30523 (11)	0.0195 (5)	
C8A	0.62787 (17)	0.28238 (15)	0.25262 (11)	0.0203 (5)	
C9A	0.64395 (17)	0.21919 (15)	0.22035 (10)	0.0189 (5)	
C10A	0.6328 (2)	0.30248 (16)	0.35591 (11)	0.0258 (6)	
H10A	0.5689	0.3114	0.3629	0.039*	
H10B	0.6638	0.3519	0.3524	0.039*	
H10C	0.6591	0.2734	0.3857	0.039*	
C11A	0.60097 (19)	0.36179 (15)	0.23513 (12)	0.0257 (6)	
H11A	0.6074	0.3662	0.1961	0.039*	
H11B	0.6397	0.3998	0.2527	0.039*	
H11C	0.5383	0.3712	0.2451	0.039*	
C12A	0.6347 (2)	0.20858 (17)	0.16140 (10)	0.0261 (6)	
H12A	0.6667	0.1619	0.1504	0.039*	
H12B	0.6603	0.2531	0.1428	0.039*	
H12C	0.5710	0.2037	0.1521	0.039*	
Co1B	0.31918 (2)	0.40924 (2)	0.373980 (14)	0.01860 (10)	
Cl1B	0.27079 (5)	0.51575 (4)	0.33151 (3)	0.02458 (16)	
Cl2B	0.22703 (5)	0.34966 (4)	0.43286 (3)	0.02729 (16)	
N1B	0.42685 (15)	0.42502 (13)	0.42153 (9)	0.0206 (5)	
N2B	0.43489 (16)	0.38675 (13)	0.46881 (8)	0.0217 (5)	
H2B	0.3908	0.3616	0.4844	0.026*	
N3B	0.34707 (15)	0.33970 (13)	0.31166 (8)	0.0194 (5)	
N4B	0.34550 (15)	0.36468 (12)	0.25999 (8)	0.0192 (5)	
H4B	0.3320	0.4119	0.2503	0.023*	
C1B	0.50868 (18)	0.45440 (15)	0.41148 (10)	0.0203 (5)	
C2B	0.56832 (18)	0.43508 (15)	0.45274 (11)	0.0211 (5)	
C3B	0.51843 (18)	0.39196 (15)	0.48875 (10)	0.0211 (6)	
C4B	0.52534 (19)	0.49953 (17)	0.36144 (11)	0.0259 (6)	
H4B1	0.5535	0.4663	0.3345	0.039*	
H4B2	0.5653	0.5428	0.3695	0.039*	
H4B3	0.4682	0.5190	0.3475	0.039*	
C5B	0.66573 (19)	0.45395 (18)	0.45800 (12)	0.0286 (6)	
H5B1	0.6763	0.4799	0.4923	0.043*	
H5B2	0.6838	0.4879	0.4285	0.043*	
H5B3	0.7011	0.4065	0.4566	0.043*	
C6B	0.5461 (2)	0.35618 (17)	0.54041 (11)	0.0283 (6)	
H6B1	0.4926	0.3404	0.5605	0.042*	
H6B2	0.5803	0.3935	0.5616	0.042*	
H6B3	0.5837	0.3111	0.5332	0.042*	
C7B	0.37141 (17)	0.26534 (15)	0.30912 (11)	0.0198 (5)	
C8B	0.38400 (18)	0.24321 (15)	0.25563 (11)	0.0204 (5)	
C9B	0.36707 (17)	0.30816 (15)	0.22547 (10)	0.0191 (5)	
C10B	0.3832 (2)	0.21733 (17)	0.35834 (11)	0.0284 (6)	
H10D	0.4472	0.2073	0.3641	0.043*	
H10E	0.3513	0.1685	0.3538	0.043*	
H10F	0.3587	0.2447	0.3894	0.043*	
C11B	0.41032 (19)	0.16500 (15)	0.23556 (11)	0.0251 (6)	
H11D	0.4018	0.1627	0.1966	0.038*	
H11E	0.3728	0.1259	0.2528	0.038*	
H11F	0.4736	0.1554	0.2441	0.038*	
C12B	0.37356 (19)	0.32148 (16)	0.16644 (10)	0.0239 (6)	
H12D	0.3412	0.3686	0.1571	0.036*	
H12E	0.3470	0.2778	0.1473	0.036*	
H12F	0.4368	0.3267	0.1562	0.036*	

### Atomic displacement parameters (Å^2^)

**Table:**

	*U*^11^	*U*^22^	*U*^33^	*U*^12^	*U*^13^	*U*^23^
Co1A	0.01982 (19)	0.01854 (19)	0.01504 (18)	0.00059 (14)	−0.00063 (13)	0.00002 (13)
Cl1A	0.0250 (3)	0.0212 (3)	0.0215 (3)	0.0030 (3)	−0.0011 (3)	−0.0038 (2)
Cl2A	0.0271 (4)	0.0282 (3)	0.0198 (3)	−0.0053 (3)	−0.0059 (3)	0.0020 (3)
N1A	0.0224 (12)	0.0221 (11)	0.0170 (11)	0.0012 (9)	−0.0023 (9)	−0.0016 (9)
N2A	0.0228 (12)	0.0264 (12)	0.0140 (11)	−0.0016 (9)	−0.0001 (9)	−0.0019 (9)
N3A	0.0226 (12)	0.0205 (11)	0.0158 (11)	0.0007 (9)	−0.0003 (9)	−0.0010 (9)
N4A	0.0189 (11)	0.0192 (11)	0.0156 (10)	0.0009 (9)	−0.0016 (8)	−0.0012 (8)
C1A	0.0212 (13)	0.0163 (12)	0.0202 (13)	0.0009 (10)	−0.0024 (10)	0.0017 (10)
C2A	0.0230 (14)	0.0222 (13)	0.0202 (13)	0.0014 (11)	−0.0001 (11)	0.0025 (11)
C3A	0.0248 (14)	0.0225 (13)	0.0197 (13)	0.0022 (11)	0.0027 (11)	0.0019 (10)
C4A	0.0224 (14)	0.0328 (16)	0.0254 (15)	0.0015 (12)	−0.0029 (12)	−0.0062 (12)
C5A	0.0255 (15)	0.0382 (17)	0.0280 (15)	−0.0046 (13)	0.0010 (12)	0.0020 (13)
C6A	0.0288 (16)	0.0378 (17)	0.0204 (14)	0.0002 (13)	0.0046 (12)	−0.0023 (12)
C7A	0.0177 (13)	0.0187 (13)	0.0222 (13)	−0.0005 (10)	0.0000 (10)	−0.0015 (10)
C8A	0.0166 (13)	0.0201 (13)	0.0241 (14)	−0.0002 (10)	−0.0010 (10)	0.0004 (11)
C9A	0.0163 (12)	0.0214 (13)	0.0191 (13)	−0.0008 (10)	−0.0023 (10)	0.0025 (10)
C10A	0.0291 (15)	0.0228 (14)	0.0255 (14)	0.0043 (12)	−0.0019 (12)	−0.0049 (11)
C11A	0.0256 (15)	0.0220 (14)	0.0295 (15)	0.0019 (11)	−0.0064 (12)	0.0038 (11)
C12A	0.0312 (16)	0.0290 (15)	0.0180 (13)	−0.0003 (12)	−0.0025 (11)	0.0044 (11)
Co1B	0.02032 (19)	0.01980 (19)	0.01568 (18)	0.00110 (14)	0.00118 (14)	−0.00009 (14)
Cl1B	0.0278 (4)	0.0231 (3)	0.0228 (3)	0.0049 (3)	0.0012 (3)	0.0036 (3)
Cl2B	0.0298 (4)	0.0324 (4)	0.0197 (3)	−0.0072 (3)	0.0068 (3)	−0.0024 (3)
N1B	0.0229 (12)	0.0228 (11)	0.0161 (11)	−0.0006 (9)	0.0005 (9)	0.0013 (9)
N2B	0.0250 (12)	0.0245 (12)	0.0155 (11)	0.0001 (9)	0.0014 (9)	0.0033 (9)
N3B	0.0211 (11)	0.0222 (11)	0.0150 (10)	0.0013 (9)	0.0015 (9)	0.0021 (9)
N4B	0.0225 (12)	0.0192 (11)	0.0158 (11)	0.0011 (9)	0.0014 (9)	0.0027 (8)
C1B	0.0222 (14)	0.0202 (13)	0.0186 (13)	0.0015 (11)	0.0012 (10)	−0.0012 (10)
C2B	0.0213 (14)	0.0213 (13)	0.0208 (13)	0.0031 (11)	−0.0003 (11)	−0.0021 (11)
C3B	0.0236 (14)	0.0219 (13)	0.0179 (13)	0.0036 (11)	−0.0004 (11)	−0.0035 (10)
C4B	0.0221 (14)	0.0305 (15)	0.0249 (15)	−0.0003 (12)	0.0014 (11)	0.0042 (12)
C5B	0.0257 (15)	0.0319 (16)	0.0282 (15)	−0.0009 (12)	−0.0024 (12)	0.0016 (12)
C6B	0.0307 (16)	0.0327 (16)	0.0216 (14)	0.0004 (13)	−0.0014 (12)	0.0023 (12)
C7B	0.0183 (13)	0.0182 (13)	0.0229 (14)	−0.0003 (10)	0.0005 (10)	−0.0006 (10)
C8B	0.0172 (13)	0.0216 (13)	0.0224 (13)	−0.0007 (10)	0.0013 (10)	−0.0017 (11)
C9B	0.0159 (13)	0.0216 (13)	0.0197 (13)	−0.0029 (10)	0.0017 (10)	−0.0039 (10)
C10B	0.0346 (17)	0.0256 (15)	0.0250 (15)	0.0044 (13)	0.0016 (12)	0.0044 (12)
C11B	0.0246 (15)	0.0208 (14)	0.0299 (15)	0.0027 (11)	0.0032 (12)	−0.0050 (11)
C12B	0.0289 (15)	0.0241 (14)	0.0188 (13)	−0.0027 (11)	0.0013 (11)	−0.0006 (11)

### Geometric parameters (Å, °)

**Table:**

Co1A—N1A	2.004 (2)	Co1B—N3B	2.012 (2)
Co1A—N3A	2.012 (2)	Co1B—N1B	2.012 (2)
Co1A—Cl1A	2.2536 (7)	Co1B—Cl1B	2.2524 (7)
Co1A—Cl2A	2.2617 (7)	Co1B—Cl2B	2.2606 (7)
N1A—C1A	1.346 (3)	N1B—C1B	1.345 (3)
N1A—N2A	1.361 (3)	N1B—N2B	1.359 (3)
N2A—C3A	1.350 (3)	N2B—C3B	1.342 (4)
N2A—H2A	0.8800	N2B—H2B	0.8800
N3A—C7A	1.345 (3)	N3B—C7B	1.345 (3)
N3A—N4A	1.366 (3)	N3B—N4B	1.359 (3)
N4A—C9A	1.352 (3)	N4B—C9B	1.345 (3)
N4A—H4A	0.8800	N4B—H4B	0.8800
C1A—C2A	1.398 (4)	C1B—C2B	1.400 (4)
C1A—C4A	1.496 (4)	C1B—C4B	1.494 (4)
C2A—C3A	1.378 (4)	C2B—C3B	1.386 (4)
C2A—C5A	1.497 (4)	C2B—C5B	1.493 (4)
C3A—C6A	1.488 (4)	C3B—C6B	1.488 (4)
C4A—H4A1	0.9800	C4B—H4B1	0.9800
C4A—H4A2	0.9800	C4B—H4B2	0.9800
C4A—H4A3	0.9800	C4B—H4B3	0.9800
C5A—H5A1	0.9800	C5B—H5B1	0.9800
C5A—H5A2	0.9800	C5B—H5B2	0.9800
C5A—H5A3	0.9800	C5B—H5B3	0.9800
C6A—H6A1	0.9800	C6B—H6B1	0.9800
C6A—H6A2	0.9800	C6B—H6B2	0.9800
C6A—H6A3	0.9800	C6B—H6B3	0.9800
C7A—C8A	1.397 (4)	C7B—C8B	1.400 (4)
C7A—C10A	1.490 (4)	C7B—C10B	1.494 (4)
C8A—C9A	1.383 (4)	C8B—C9B	1.380 (4)
C8A—C11A	1.503 (4)	C8B—C11B	1.502 (4)
C9A—C12A	1.487 (4)	C9B—C12B	1.493 (4)
C10A—H10A	0.9800	C10B—H10D	0.9800
C10A—H10B	0.9800	C10B—H10E	0.9800
C10A—H10C	0.9800	C10B—H10F	0.9800
C11A—H11A	0.9800	C11B—H11D	0.9800
C11A—H11B	0.9800	C11B—H11E	0.9800
C11A—H11C	0.9800	C11B—H11F	0.9800
C12A—H12A	0.9800	C12B—H12D	0.9800
C12A—H12B	0.9800	C12B—H12E	0.9800
C12A—H12C	0.9800	C12B—H12F	0.9800
			
N1A—Co1A—N3A	110.94 (9)	N3B—Co1B—N1B	111.86 (9)
N1A—Co1A—Cl1A	112.73 (7)	N3B—Co1B—Cl1B	101.42 (6)
N3A—Co1A—Cl1A	100.38 (6)	N1B—Co1B—Cl1B	114.79 (7)
N1A—Co1A—Cl2A	101.96 (7)	N3B—Co1B—Cl2B	110.52 (7)
N3A—Co1A—Cl2A	112.31 (7)	N1B—Co1B—Cl2B	99.43 (7)
Cl1A—Co1A—Cl2A	118.84 (3)	Cl1B—Co1B—Cl2B	119.22 (3)
C1A—N1A—N2A	105.5 (2)	C1B—N1B—N2B	105.6 (2)
C1A—N1A—Co1A	132.03 (18)	C1B—N1B—Co1B	131.60 (18)
N2A—N1A—Co1A	121.63 (17)	N2B—N1B—Co1B	120.93 (17)
C3A—N2A—N1A	111.3 (2)	C3B—N2B—N1B	111.7 (2)
C3A—N2A—H2A	124.3	C3B—N2B—H2B	124.2
N1A—N2A—H2A	124.3	N1B—N2B—H2B	124.2
C7A—N3A—N4A	105.7 (2)	C7B—N3B—N4B	105.5 (2)
C7A—N3A—Co1A	132.68 (18)	C7B—N3B—Co1B	132.09 (18)
N4A—N3A—Co1A	121.65 (16)	N4B—N3B—Co1B	122.38 (16)
C9A—N4A—N3A	111.3 (2)	C9B—N4B—N3B	111.6 (2)
C9A—N4A—H4A	124.4	C9B—N4B—H4B	124.2
N3A—N4A—H4A	124.4	N3B—N4B—H4B	124.2
N1A—C1A—C2A	110.3 (2)	N1B—C1B—C2B	110.3 (2)
N1A—C1A—C4A	120.5 (2)	N1B—C1B—C4B	120.4 (2)
C2A—C1A—C4A	129.2 (2)	C2B—C1B—C4B	129.3 (2)
C3A—C2A—C1A	105.7 (2)	C3B—C2B—C1B	105.4 (2)
C3A—C2A—C5A	125.7 (3)	C3B—C2B—C5B	125.7 (3)
C1A—C2A—C5A	128.6 (3)	C1B—C2B—C5B	128.9 (3)
N2A—C3A—C2A	107.1 (2)	N2B—C3B—C2B	107.1 (2)
N2A—C3A—C6A	123.3 (3)	N2B—C3B—C6B	123.3 (3)
C2A—C3A—C6A	129.6 (3)	C2B—C3B—C6B	129.7 (3)
C1A—C4A—H4A1	109.5	C1B—C4B—H4B1	109.5
C1A—C4A—H4A2	109.5	C1B—C4B—H4B2	109.5
H4A1—C4A—H4A2	109.5	H4B1—C4B—H4B2	109.5
C1A—C4A—H4A3	109.5	C1B—C4B—H4B3	109.5
H4A1—C4A—H4A3	109.5	H4B1—C4B—H4B3	109.5
H4A2—C4A—H4A3	109.5	H4B2—C4B—H4B3	109.5
C2A—C5A—H5A1	109.5	C2B—C5B—H5B1	109.5
C2A—C5A—H5A2	109.5	C2B—C5B—H5B2	109.5
H5A1—C5A—H5A2	109.5	H5B1—C5B—H5B2	109.5
C2A—C5A—H5A3	109.5	C2B—C5B—H5B3	109.5
H5A1—C5A—H5A3	109.5	H5B1—C5B—H5B3	109.5
H5A2—C5A—H5A3	109.5	H5B2—C5B—H5B3	109.5
C3A—C6A—H6A1	109.5	C3B—C6B—H6B1	109.5
C3A—C6A—H6A2	109.5	C3B—C6B—H6B2	109.5
H6A1—C6A—H6A2	109.5	H6B1—C6B—H6B2	109.5
C3A—C6A—H6A3	109.5	C3B—C6B—H6B3	109.5
H6A1—C6A—H6A3	109.5	H6B1—C6B—H6B3	109.5
H6A2—C6A—H6A3	109.5	H6B2—C6B—H6B3	109.5
N3A—C7A—C8A	110.2 (2)	N3B—C7B—C8B	110.2 (2)
N3A—C7A—C10A	121.4 (2)	N3B—C7B—C10B	122.0 (2)
C8A—C7A—C10A	128.4 (2)	C8B—C7B—C10B	127.7 (2)
C9A—C8A—C7A	106.0 (2)	C9B—C8B—C7B	105.6 (2)
C9A—C8A—C11A	127.5 (2)	C9B—C8B—C11B	127.5 (2)
C7A—C8A—C11A	126.6 (2)	C7B—C8B—C11B	127.0 (2)
N4A—C9A—C8A	106.8 (2)	N4B—C9B—C8B	107.1 (2)
N4A—C9A—C12A	122.0 (2)	N4B—C9B—C12B	122.2 (2)
C8A—C9A—C12A	131.1 (2)	C8B—C9B—C12B	130.7 (2)
C7A—C10A—H10A	109.5	C7B—C10B—H10D	109.5
C7A—C10A—H10B	109.5	C7B—C10B—H10E	109.5
H10A—C10A—H10B	109.5	H10D—C10B—H10E	109.5
C7A—C10A—H10C	109.5	C7B—C10B—H10F	109.5
H10A—C10A—H10C	109.5	H10D—C10B—H10F	109.5
H10B—C10A—H10C	109.5	H10E—C10B—H10F	109.5
C8A—C11A—H11A	109.5	C8B—C11B—H11D	109.5
C8A—C11A—H11B	109.5	C8B—C11B—H11E	109.5
H11A—C11A—H11B	109.5	H11D—C11B—H11E	109.5
C8A—C11A—H11C	109.5	C8B—C11B—H11F	109.5
H11A—C11A—H11C	109.5	H11D—C11B—H11F	109.5
H11B—C11A—H11C	109.5	H11E—C11B—H11F	109.5
C9A—C12A—H12A	109.5	C9B—C12B—H12D	109.5
C9A—C12A—H12B	109.5	C9B—C12B—H12E	109.5
H12A—C12A—H12B	109.5	H12D—C12B—H12E	109.5
C9A—C12A—H12C	109.5	C9B—C12B—H12F	109.5
H12A—C12A—H12C	109.5	H12D—C12B—H12F	109.5
H12B—C12A—H12C	109.5	H12E—C12B—H12F	109.5
			
N3A—Co1A—N1A—C1A	60.6 (3)	N3B—Co1B—N1B—C1B	59.8 (3)
Cl1A—Co1A—N1A—C1A	−51.1 (2)	Cl1B—Co1B—N1B—C1B	−55.1 (3)
Cl2A—Co1A—N1A—C1A	−179.6 (2)	Cl2B—Co1B—N1B—C1B	176.5 (2)
N3A—Co1A—N1A—N2A	−107.49 (19)	N3B—Co1B—N1B—N2B	−101.98 (19)
Cl1A—Co1A—N1A—N2A	140.83 (17)	Cl1B—Co1B—N1B—N2B	143.17 (17)
Cl2A—Co1A—N1A—N2A	12.3 (2)	Cl2B—Co1B—N1B—N2B	14.73 (19)
C1A—N1A—N2A—C3A	0.9 (3)	C1B—N1B—N2B—C3B	0.6 (3)
Co1A—N1A—N2A—C3A	171.76 (17)	Co1B—N1B—N2B—C3B	166.59 (17)
N1A—Co1A—N3A—C7A	63.9 (3)	N1B—Co1B—N3B—C7B	63.7 (3)
Cl1A—Co1A—N3A—C7A	−176.7 (2)	Cl1B—Co1B—N3B—C7B	−173.5 (2)
Cl2A—Co1A—N3A—C7A	−49.4 (3)	Cl2B—Co1B—N3B—C7B	−46.1 (3)
N1A—Co1A—N3A—N4A	−115.55 (19)	N1B—Co1B—N3B—N4B	−114.79 (19)
Cl1A—Co1A—N3A—N4A	3.8 (2)	Cl1B—Co1B—N3B—N4B	8.0 (2)
Cl2A—Co1A—N3A—N4A	131.07 (17)	Cl2B—Co1B—N3B—N4B	135.43 (18)
C7A—N3A—N4A—C9A	0.6 (3)	C7B—N3B—N4B—C9B	0.7 (3)
Co1A—N3A—N4A—C9A	−179.83 (17)	Co1B—N3B—N4B—C9B	179.50 (17)
N2A—N1A—C1A—C2A	−0.3 (3)	N2B—N1B—C1B—C2B	−0.4 (3)
Co1A—N1A—C1A—C2A	−169.78 (19)	Co1B—N1B—C1B—C2B	−164.27 (19)
N2A—N1A—C1A—C4A	178.7 (2)	N2B—N1B—C1B—C4B	178.9 (2)
Co1A—N1A—C1A—C4A	9.2 (4)	Co1B—N1B—C1B—C4B	15.0 (4)
N1A—C1A—C2A—C3A	−0.4 (3)	N1B—C1B—C2B—C3B	0.1 (3)
C4A—C1A—C2A—C3A	−179.3 (3)	C4B—C1B—C2B—C3B	−179.1 (3)
N1A—C1A—C2A—C5A	179.4 (3)	N1B—C1B—C2B—C5B	178.8 (3)
C4A—C1A—C2A—C5A	0.5 (5)	C4B—C1B—C2B—C5B	−0.4 (5)
N1A—N2A—C3A—C2A	−1.2 (3)	N1B—N2B—C3B—C2B	−0.6 (3)
N1A—N2A—C3A—C6A	179.5 (2)	N1B—N2B—C3B—C6B	179.3 (2)
C1A—C2A—C3A—N2A	0.9 (3)	C1B—C2B—C3B—N2B	0.3 (3)
C5A—C2A—C3A—N2A	−178.9 (3)	C5B—C2B—C3B—N2B	−178.5 (3)
C1A—C2A—C3A—C6A	−179.8 (3)	C1B—C2B—C3B—C6B	−179.6 (3)
C5A—C2A—C3A—C6A	0.4 (5)	C5B—C2B—C3B—C6B	1.6 (5)
N4A—N3A—C7A—C8A	−0.7 (3)	N4B—N3B—C7B—C8B	−0.8 (3)
Co1A—N3A—C7A—C8A	179.77 (19)	Co1B—N3B—C7B—C8B	−179.47 (19)
N4A—N3A—C7A—C10A	178.1 (2)	N4B—N3B—C7B—C10B	178.4 (2)
Co1A—N3A—C7A—C10A	−1.4 (4)	Co1B—N3B—C7B—C10B	−0.2 (4)
N3A—C7A—C8A—C9A	0.6 (3)	N3B—C7B—C8B—C9B	0.7 (3)
C10A—C7A—C8A—C9A	−178.1 (3)	C10B—C7B—C8B—C9B	−178.5 (3)
N3A—C7A—C8A—C11A	−178.6 (2)	N3B—C7B—C8B—C11B	−179.3 (2)
C10A—C7A—C8A—C11A	2.7 (5)	C10B—C7B—C8B—C11B	1.6 (5)
N3A—N4A—C9A—C8A	−0.2 (3)	N3B—N4B—C9B—C8B	−0.3 (3)
N3A—N4A—C9A—C12A	−177.4 (2)	N3B—N4B—C9B—C12B	−177.7 (2)
C7A—C8A—C9A—N4A	−0.2 (3)	C7B—C8B—C9B—N4B	−0.2 (3)
C11A—C8A—C9A—N4A	178.9 (3)	C11B—C8B—C9B—N4B	179.7 (3)
C7A—C8A—C9A—C12A	176.6 (3)	C7B—C8B—C9B—C12B	176.9 (3)
C11A—C8A—C9A—C12A	−4.2 (5)	C11B—C8B—C9B—C12B	−3.2 (5)

### Hydrogen-bond geometry (Å, °)

**Table:**

*D*—H···*A*	*D*—H	H···*A*	*D*···*A*	*D*—H···*A*
N2A—H2A···Cl2B^i^	0.88	2.55	3.290 (2)	142.
N2A—H2A···Cl2A	0.88	2.86	3.373 (2)	118.
N4A—H4A···Cl1B^ii^	0.88	2.61	3.416 (2)	152.
N4A—H4A···Cl1A	0.88	2.79	3.309 (2)	119.
N2B—H2B···Cl2A^iii^	0.88	2.70	3.456 (2)	145.
N2B—H2B···Cl2B	0.88	2.76	3.286 (2)	119.
N4B—H4B···Cl1A^iv^	0.88	2.57	3.363 (2)	150.
N4B—H4B···Cl1B	0.88	2.86	3.365 (2)	118.

Symmetry codes: (i) *x*+1/2, −*y*+1/2, −*z*+1; (ii) −*x*+1, *y*−1/2, −*z*+1/2; (iii) *x*−1/2, −*y*+1/2, −*z*+1; (iv) −*x*+1, *y*+1/2, −*z*+1/2.
